# Single ChAdOx1 nCoV‐19 dose elicits stronger immune response in previously infected individuals than in SARS‐CoV2 naive persons

**DOI:** 10.1002/iid3.1159

**Published:** 2024-01-19

**Authors:** Fawzi Ebrahim, Asma Alboueishi, Inas M. Alhudiri, Salah Al Tabal, Yosra Lamami, Samira Al Dwigen, Sondos Ashleb, Noha Ejenfawi, Mohamed B. Milad, Hayat Rhoumah, Salah Edin El Meshri, Adam Elzagheid

**Affiliations:** ^1^ Departement/Molecular Diagnostic Group, Cells and Tissue Culture Libyan Biotechnology Research Center Tripoli Libya

**Keywords:** ChAdOx1 (AstraZeneca), COVID‐19 infection, IgG antibodies, previous infection, SARS‐CoV‐2

## Abstract

**Background:**

Current vaccines against COVID‐19 effectively reduce morbidity and mortality and are vitally important for controlling the pandemic. Between December 2020 and February 2021, adenoviral vector vaccines such as ChAdOx1 (AstraZeneca‐Oxford) were put in use. Recent reports demonstrate robust serological responses to a single dose of messenger RNA vaccines in individuals previously infected with SARS‐CoV‐2. We aimed to study the association between previous COVID‐19 infection and antibody levels after a single dose of ChAdOx1 nCoV‐19.

**Methods:**

This cross‐sectional study was conducted on 657 individuals who were either convalescent or SARS‐CoV‐2 naive and had received one dose of ChAdOx1 (AstraZeneca). A questionnaire was used to collect data on age, sex, and self‐reported history of COVID‐19 infection. We then compared the average levels of immunoglobulin G (IgG) between the previously infected and COVID‐19‐naive participants.

**Results:**

We compared the antibody responses of individuals with confirmed prior COVID‐19 infection with those of individuals without prior evidence of infection. The mean antibody levels in those who reported no history of COVID‐19 infection were substantially lower than in those who were previously infected, in both males and females. Sex‐related differences were observed when we compared antibody levels between men and women. In males, anti‐S IgG antibody levels were higher in those who had been previously infected (156.1 vs. 87.69 AU/mL, *p* = .009), compared with the same pattern was observed in females (113.5 vs. 90.69 AU/mL, *p* = .005).

**Conclusions:**

Previous COVID‐19 infection is associated with higher levels of SARS‐CoV‐2 antibodies following ChAdOx1 (AstraZeneca) vaccination. Our finding supports the notion that a single dose of ChAdOx1 nCoV‐19 administered post‐SARS‐CoV‐2 infection serves as an effective immune booster. This provides a possible rationale for a single‐dose vaccine regimen for previously infected individuals.

## Graphical abstract

Previous COVID‐19 infection is associated with higher levels of SARS‐CoV‐2 antibodies following ChAdOx1 (AstraZeneca) vaccination. single dose of ChAdOx1 nCoV‐19 administered post‐SARS‐CoV‐2 infection serves as an effective immune booster

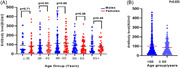

## 1 INTRODUCTION

Vaccines to prevent COVID‐19 infection are crucial for an effective global pandemic response against confirmed COVID‐19 symptoms, reducing hospital admissions and mortality.^1^ Widespread morbidity and mortality associated with the COVID‐19 pandemic precipitated the most extensive and rapid global vaccine development program in history.^2^ The World Health Organization and the US Food and Drug Administration (FDA) approved some COVID‐19 vaccines in September 2020, of which ChAdOx1 nCoV‐19 (AZD1222) is one of the most common. This is a chimpanzee adenoviral‐vectored vaccine with a full‐length SARS‐CoV‐2 spike insert. ChAdOx1 nCoV‐19 has been shown in clinical trials to be effective in greatly reducing symptomatic COVID‐19, hospitalization, and death. The safety and immunogenicity of the vaccine were assessed in four randomized controlled trials in the United Kingdom, Brazil, and South Africa.^3^ ChAdOx1 nCoV‐19 was granted emergency use authorization for adults by the UK Medicines and Healthcare Products Regulatory Agency on December 30, 2020, with a regimen of two doses with an interval of 4–12 weeks for adults aged 18 years and older. It has since been authorized for use in many other countries.^4^

According to the National Center for Disease Control in Libya, more than three million people have been vaccinated, of whom >885,000 had received ChAdOx1 nCoV‐19. There are very limited published data on AstraZeneca vaccine effectiveness in Libya and most Middle Eastern countries.^5^

Vaccination after recovery from natural SARS‐CoV‐2 infection, or “hybrid immunity,” has been reported to substantially increase both the potency and breadth of the humoral response to SARS‐CoV‐2.^6^ Recent data by Havervall et al. suggest that administration of the first adenovector vaccine dose in individuals who had recovered from COVID‐19 induces an immune response that is equal to or stronger than that induced in SARS‐CoV‐2 naive individuals following the second messenger RNA vaccine dose. Such findings challenge the current vaccine guidelines recommending a two‐dose regimen regardless of pre‐existing immunity.^7^

This study investigated the association between previous COVID‐19 infection and antibody levels after a single dose of the ChAdOx1 COVID‐19 vaccine.

## 2 MATERIALS AND METHODS

### 2.1 Participants and ethics statement

Participants were recruited from people visiting healthcare centers and employees in state institutions in several cities in northern Libya. The recruitment period started from August 2021 to December 2021. The study recruited people who had received one dose of ChAdOx1. The inclusion criteria were having received one dose of ChAdOx1 with or without previous infection and age >18 years. The exclusion criteria were pregnancy, blood, or plasma transfusion during the 3 months preceding the study, immunosuppressive therapy, recent chemotherapy, autoimmune diseases, and renal dialysis.

The Bioethics Committee at the Libyan Biotechnology Research Center in Tripoli, Libya (Ref No. BEC‐BTRC 8‐2020) approved the study. The study protocol was compatible with the World Medical Association Declaration of Helsinki (Ethical Principles for Medical Research Involving Human Subjects). All participants provided written informed consent to participate. Those who agreed to participate were given an information sheet detailing the study aim, pledging anonymity of their information, and explaining that they have the right to withdraw from the study at any time.

### 2.2 Data collection

A self‐administered questionnaire was used to collect data on sex, age, the type of vaccines received, the date(s) of vaccination, side effects, severity of symptoms, previous COVID‐19 (defined as confirmed SARS CoV‐2 infection by either polymerase chain reaction or rapid antigen tests before vaccination) and whether the infection (if there was one) was before or after receiving the vaccine (breakthrough infection). Breakthrough infections were not counted as previous infection. Information on past medical history and influenza vaccination status were also noted. Partially completed questionnaires that did not contain information on vaccination status and COVID‐19 infection status were excluded from further analysis. In addition, individuals who fit the exclusion criteria were also excluded from the data analysis.

To address the possibility of recall bias or over‐ or understatements by participants, the interview questionnaire included specific questions aimed at capturing relevant details of participants' prior COVID‐19 infection. The questions were formulated in a neutral and unbiased manner, avoiding leading or suggestive language that could potentially influence participants' responses. The interviewers were trained extensively to ensure consistency in administering the questionnaire and to maintain a standardized approach across all participants.

Furthermore, we took steps to validate the self‐reported history of COVID‐19 infection. The self‐reports provided by participants were cross‐checked with available laboratory tests, and other objective sources of information wherever possible. This validation process allowed us to assess the concordance between self‐reports and verified data, providing additional confidence in the accuracy of the participants' accounts.

### 2.3 Determination of antibody levels

Blood samples of 4 mL were collected in plain (no additive) Vacutainer tubes. The samples were coded and centrifuged at ×2000 *g* for 10 min at room temperature to separate the serum, which was stored at −20°C until analyzed within 48 h.

A Beckman Coulter Access Anti‐SARS‐CoV‐2 immunoglobulin G (IgG) assay was used on a UniCel Dxl 6 Access Immunoassay System to determine anti‐SARS‐CoV‐2 antibody levels according to the manufacturer's instructions (Beckman Coulter). A sample was considered reactive (positive) for anti‐S IgG if the result was ≥10 AU/mL.

### 2.4 Statistical analysis

A web application was developed with PHP, MySQL, and JavaScript specifically for electronically collecting survey data and initial statistical analysis. However, the statistical analysis was performed using Microsoft Excel and GraphPad Prism version 9.3. The descriptive statistics included mean, standard deviation, and percentages. Mean IgG levels were plotted at different time intervals (in weeks) between the date of serum collection and the date of vaccination. The differences between mean values were compared by the unpaired Student's *t* test. *p* < .05 were considered statistically significant.

## 3 RESULTS

### 3.1 Characteristics of the study groups

**Figure 1:**
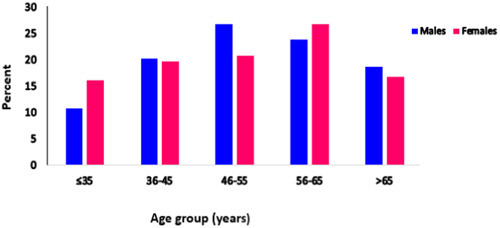
The distribution of the participants by sex and age. Data and blood sample were obtained from 657 participants who had received one dose of ChAdOx1 (353 were male and 304 were female). According to study design criteria all participants involved in the study were among ≤35 and >65 years.

The study recruited 657 individuals who had received one dose of ChAdOx1: 353 males aged from 20 to 88 and 304 females aged from 30 to 90 years. Their sex and age distributions are presented in Figure 1.

Most of the participants (556, 85%) were naive to SARS‐CoV‐2 and 101 (15%) were convalescent. In the convalescent group, most of the infections were mild (71.3%). Moderate and severe infections represented 17.8% and 10.9% of the reported symptomatic infections, respectively.

### 3.2 Antibody levels

**Figure 2:**
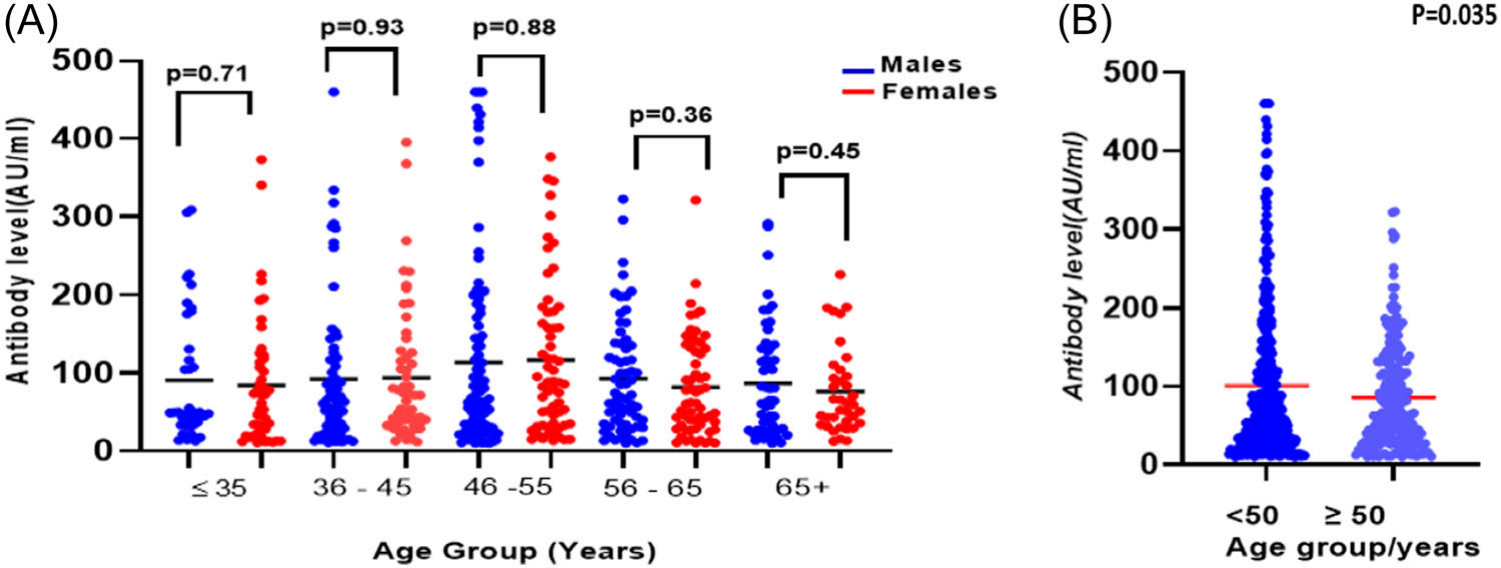
(A) Immunoglobulin G (IgG) levels against the Spike protein in the different sex and age groups in naïve individuals. There was no statistically significant difference between males and females in the different age groups. Red color = female, blue color = male. Black line represents the mean antibody level for each cohort. (B) Comparison of IgG Levels against the Spike protein in naïve individuals younger than 50 years and older than 50 years. Black line intersecting scatter plots represents the mean IgG levels.

The mean level of IgG in vaccinated females (104 AU/mL) compared with vaccinated males (94.09 AU/mL), was not significantly different (*p* = .286). Since a higher proportion of previously infected individuals was observed in the elderly group compared to the younger age group (55.8%), the distributions of antibody titers in the different sex and age groups were analyzed in individuals with no previous history of COVID‐19 to overcome any bias (Figure 2A). There were no significant differences between males and females in the different age groups (*p* = .1892), but the antibody levels were significantly higher in those <50 years compared with older participants (Figure 2B).

### 3.3 Comparison of total anti‐SARS‐CoV‐2 antibody levels in vaccinated individuals with or without a history of COVID‐19 infection

**Figure 3:**
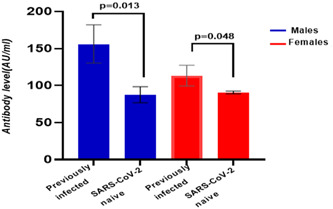
Comparison of anti‐SARS‐CoV‐2 antibody levels in vaccinated individuals in the presence or absence of a history of COVID‐19 infection by sex. The level of SARS‐CoV‐2 immunoglobulin G antibodies in previously infected was significantly higher than those with no prior infection.

We compared the mean levels of anti‐SARS‐CoV‐2 IgG antibodies in the vaccinated individuals based on age, sex, and history of SARS‐CoV‐2 infection, as well as the severity of disease symptoms. In both sexes, the mean level of SARS‐CoV‐2 IgG antibodies in those who had been previously infected was significantly higher than those who had not been infected (Figure 3).

### 3.4 Comparison of total anti‐SARS‐CoV‐2 antibody levels in vaccinated individuals according to severity of infection

**Figure 4:**
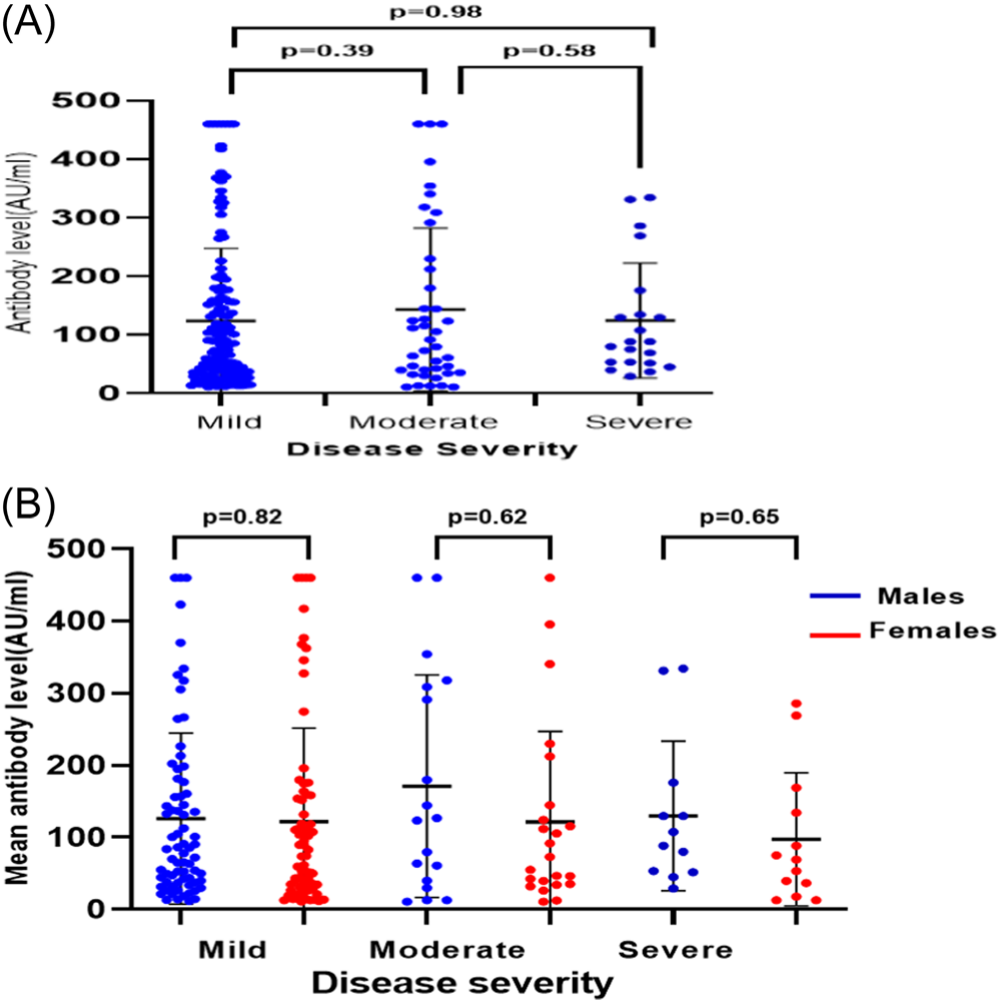
(A) Total anti‐SARS‐CoV‐2 antibody levels in vaccinated participants (single dose and previously infected). The black line intersecting scatter plots represents the mean immunoglobulin G (IgG) levels. There is no significant difference in antibody response between the disease severity statuses. The *p* value between mild versus moderate was *p* = .39, mild versus severe *p* = .98, and moderate versus severe *p* = .58. (B) Total anti‐SARS‐CoV‐2 antibody levels in previously infected males and females by disease severity. The level of IgG was not statistically different in the disease severity groups between genders. The *p* value was .82, .62, and .65 in mild, moderate, and severe disease respectively as shown in the figure.

For vaccinated individuals with a history of COVID‐19, IgG levels did not vary significantly between different disease severity groups (*p* = .835) (Figure 4A). Among vaccinated males who had a previous COVID‐19 infection, the mean level of IgG was not statistically different in the disease severity groups in both males (*p* = .851) and females (*p* = .739) (Figure 4B).

## 4 DISCUSSION

Considerable research on vaccine‐elicited anti‐SARS‐CoV‐2 antibody profiles has been done since the beginning of the pandemic. Antibody levels have been assessed in relation to the interval between the first and the second dose of the vaccine, as well as demographic factors and history of COVID‐19 infection.^8^ The present study holds unique significance due to its timely examination of seropositivity in Libyan patients, a country with limited prior investigations. In Hung et al. provided evidence that a single dose of ChAdOx1 nCoV‐19 is highly effective in the 90 days after vaccination, and that a longer prime–boost interval results in stronger protection against symptomatic COVID‐19.^9^ In addition, Bernal et al. demonstrated that a single dose of the ChAdOx1‐S vaccine was about 60%–75% effective against symptomatic disease and provided an additional protective effect against hospital admission.^10^

Our findings show that following natural infection, a single dose of this vaccine elicits a robust serological response against SARS‐CoV‐2. The average antibody level in individuals previously infected with COVID‐19 was significantly higher in males and in females than in those without previous infection. A significant difference has been reported between persons with or without a history of COVID‐19 infection.^11^ Similar published observations were also made by our team at the Libyan Biotechnology Research Center.^5^

Individuals younger than 50 years exhibited higher antibody titers compared to those older than 50 years. A study by Wolszczak et al. demonstrated a significant age‐related difference in antibody levels among individuals who had received one dose of the COVID‐19 vaccine. They found that the level of total anti‐SARS‐CoV‐2 antibodies in patients with a history of COVID‐19 was higher in those aged <50 years in comparison to those >50 years old.^12^ These findings suggest that younger individuals may have a more robust immune response to COVID‐19 vaccination.^13^

In many studies, the first vaccine dose of ChAdOx1 nCoV‐19 triggers a strong humoral immune response.^14^, ^15^ Our study confirms these observations. Moreover, in the group with previous COVID‐19 infection, the total anti‐SARS‐CoV‐2S antibody levels were significantly higher in men than in women (*p* < .009). This observation points to a relationship between sex and humoral immune response to vaccination.^16^

Our study found that antibody levels in vaccinated convalescents does not correlate with disease severity, with no gender‐based differences observed in antibody responses. This is consistent with other research, Legros et al. found no significant difference in anti‐SARS‐CoV‐2 IgG levels between patients with mild, moderate, and severe COVID‐19, but the neutralizing antibody response was found to correlate with disease severity.^17^

This study has some limitations. First, we acknowledge the inherent limitations of relying on self‐reported data, including the possibility of recall bias and participants' potential misinterpretation of the questions. These limitations are important to consider when interpreting our findings. However, we believe that the measures we implemented, such as the interview questionnaire design, training of interviewers, and validation procedures, have effectively addressed these concerns and strengthened the validity of our study's findings.

Our study employed a binding antibody assay to assess humoral immune responses in vaccinated convalescents. While this approach provides valuable insights into antibody levels, it has limitations in understanding the functional capacity of these antibodies to neutralize the virus. This distinction is crucial, as neutralizing antibodies are considered the primary drivers of protection against severe COVID‐19.

Therefore, the lack of observed correlation between our measured antibody levels and disease severity should be interpreted with caution. Neutralizing antibody responses, not captured by our assay, might hold greater explanatory power in this regard. This possibility is supported by studies like Cantoni et al., who demonstrated a significant association between neutralizing antibody titers and disease severity using a standardized WHO assay.^18^

## 5 CONCLUSION

In individuals previously infected with SARS‐CoV‐2, a single dose of ChAdOx1 nCoV‐19 seems to act as a booster of the antibody response triggered by the infection, leading to a stronger humoral antibody response than a single dose in individuals not previously exposed to the virus. This provides a possible rationale for a single‐dose vaccine regimen for previously infected individuals.

## AUTHOR CONTRIBUTIONS

Adam Elzagheid, Fawzi Ebrahim, and Inas.M. Alhudiri contributed study conception and design. Salah Altabal, Samira Al Dwigen, Sondos Ashleb, NohaEjenfawi, Mohamed B. Milad, and Hayat Rhoumah analyzed the data. Asma Alboueishi wrote the introduction. Yosra Lammami wrote the methods. Asma Alboueishi and Fawzi Ebrahim wrote the results and discussion. Salah Edin El Meshri contributed review and editing. All authors had full access to all the data in the study, reviewed the final manuscript, and approved its submission for publication.

## CONFLICT OF INTEREST STATEMENT

The authors declare no conflict of interest.

## Data Availability

The data are available upon reasonable request.
